# Supplementary material for: Time-reversal symmetry breaking type-II Weyl state in YbMnBi2
Source: Nat Commun. 2019 Jul 31;10:3424. doi: 10.1038/s41467-019-11393-5 (PMC6668437; doi:10.1038/s41467-019-11393-5)
Supplement: Supplementary file 1 — Supplementary Information [file 41467_2019_11393_MOESM1_ESM.pdf]

Time-reversal symmetry breaking type-II Weyl state in $YbMnBi_2$

Borisenko et al.

Supplementary Figures

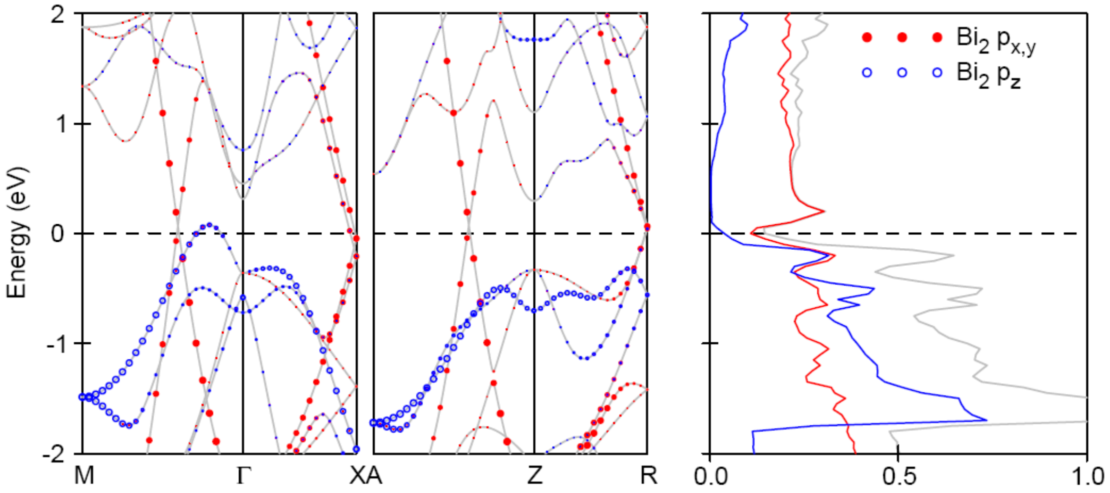

Supplementary Figure 1. Calculations of $YbMnBi_2$ without SOC, Yb 4f and Mn d states. Bi_2 are the bismuth atoms from the 2D network. Yb $4f^{14}$ and Mn $3d^5$ states are treated as quasi-core states. As expected, bands at EF are formed by bismuth 2 p states from the two-dimensional networks. There are clear Dirac-like crossings along Γ(Z)–M(A) and Γ (Z)–X(R) near X.

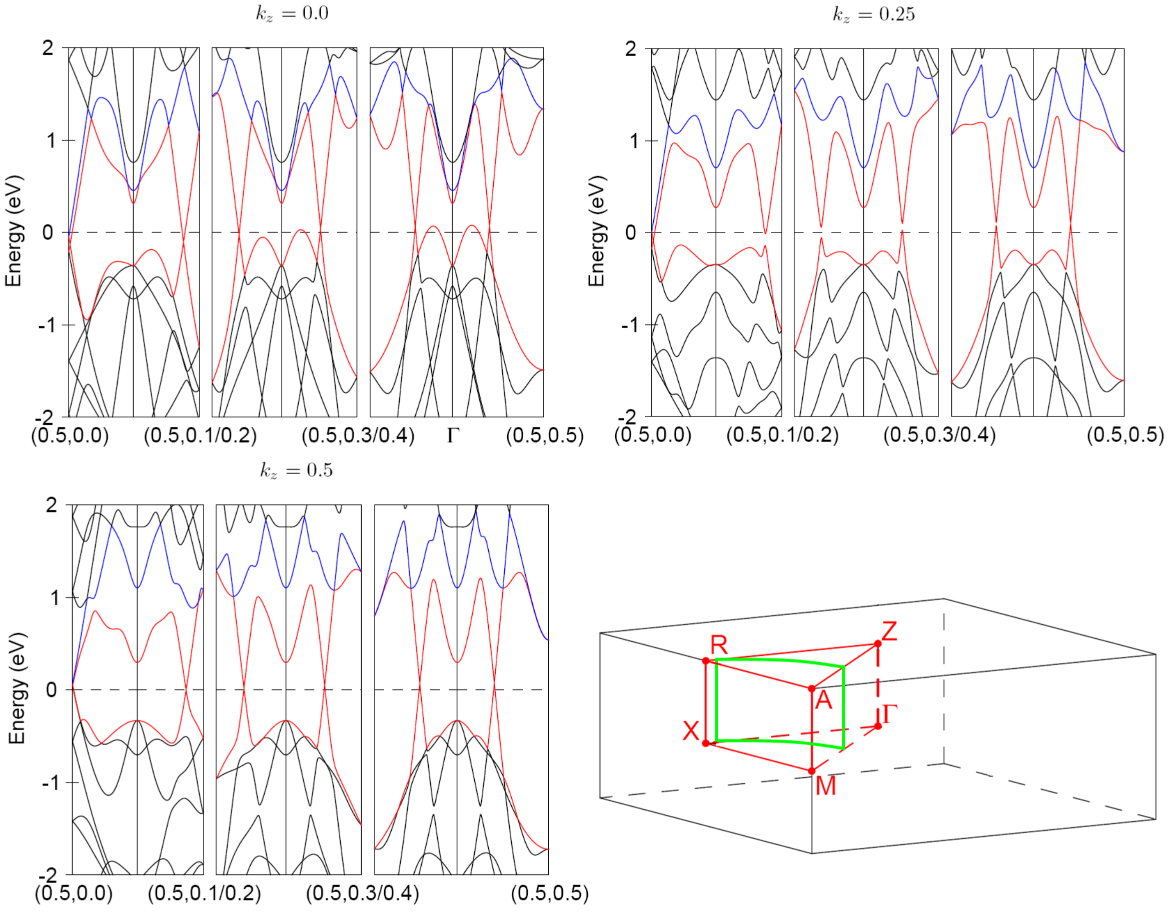

Supplementary Figure 2. Bands of $YbMnBi_2$ calculated along $(0,0,k_z)$–$(0.5,k_y,k_z)$ lines with k_y = 0,0.1, . . . 0.5, and k_z = 0, 0.25, 0.5. Green loop in the irreducible part of the BZ shows the locus of Dirac-like crossings. The crossings along $(0,0,k_z)$–$(0.5,0,k_z)$ and $(0,0,k_z)$–$(0.5,0.5,k_z)$ lines survive for arbitrary k_z. Thus, they should be protected by vertical glide mirror planes M_x and M_{xy}, respectively. Crossings for k_z=0, 0.5 and for an arbitrary k_y should be protected by a horizontal glide mirror plane M_z.

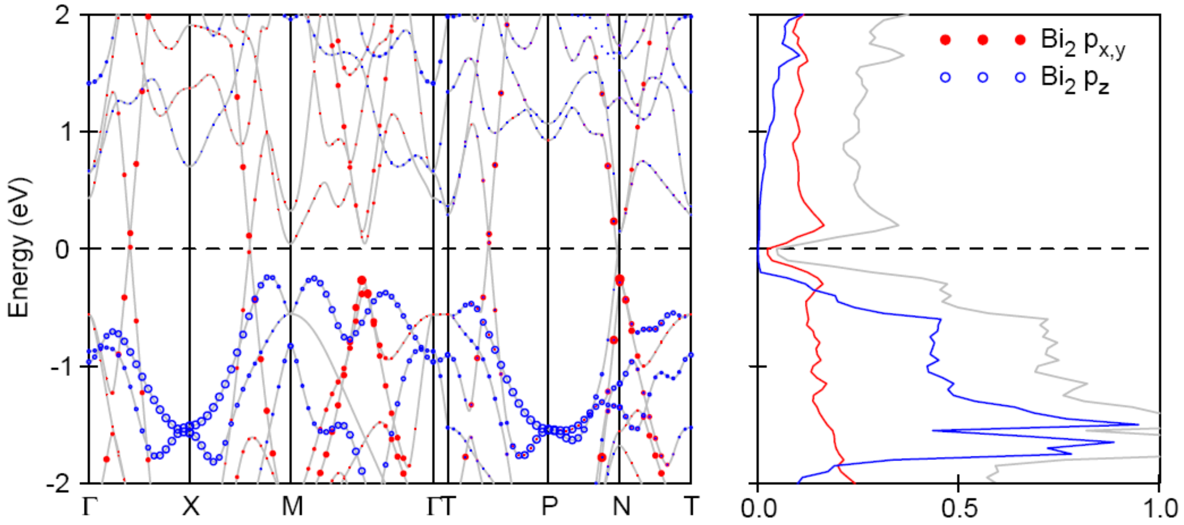

Supplementary Figure 3. Calculations of $EuMnBi_2$ without SOC, Eu 4f and Mn d states. Bi_2 are the bismuth atoms from the 2D network.

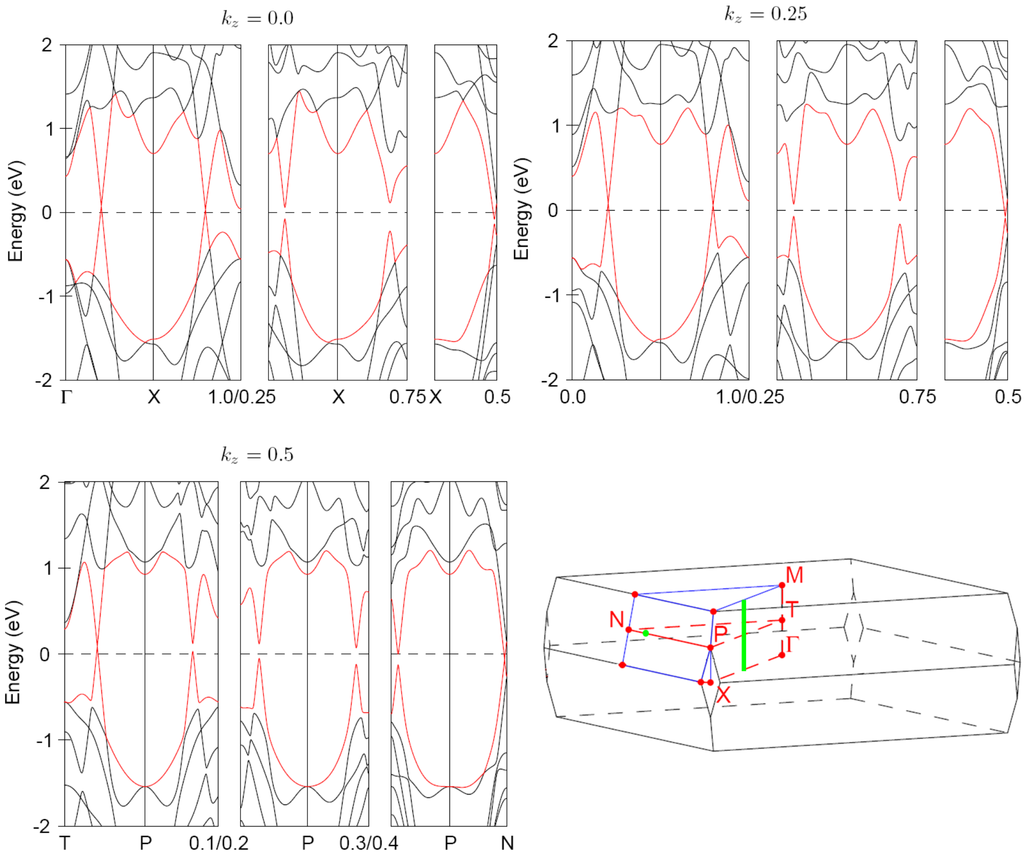

Supplementary Figure 4. Bands of $EuMnBi_2$ calculated along $(0.5,0.5,k_z)$–$(k_x,0,k_z)$ lines with k_x = 0,0.25,0.5,0.75 and k_z = 0, 0.25 and along P – $(k_x,0,0.5)$ with k_x = 0,0.1,0.2,0.3,0.4,0.5. Green line shows the locus of Dirac-like crossings. Green point is a 3D Dirac point. In this case linear dispersions cross only in a vertical Γ–M–P–X plane and along N–P line creating a nodal line and 3D Dirac point near N-point.

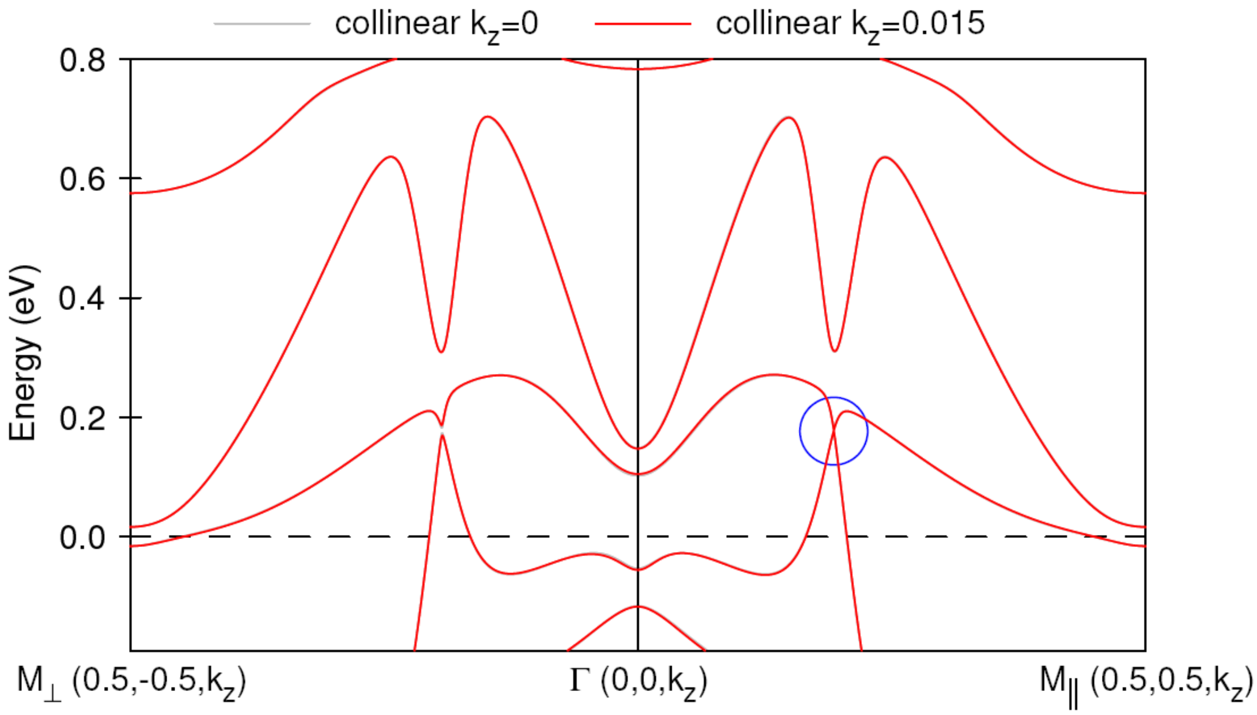

Supplementary Figure 5. 3D-Dirac point at (0.193,0.193,0.015) in $YbMnBi_2$.Upon inclusion of SOC and AFM into the computational scheme all Dirac crossings become gapped in $EuMnBi_2$. In contrast, in $YbMnBi_2$ four 3D-Dirac points are observed. One of them is shown.

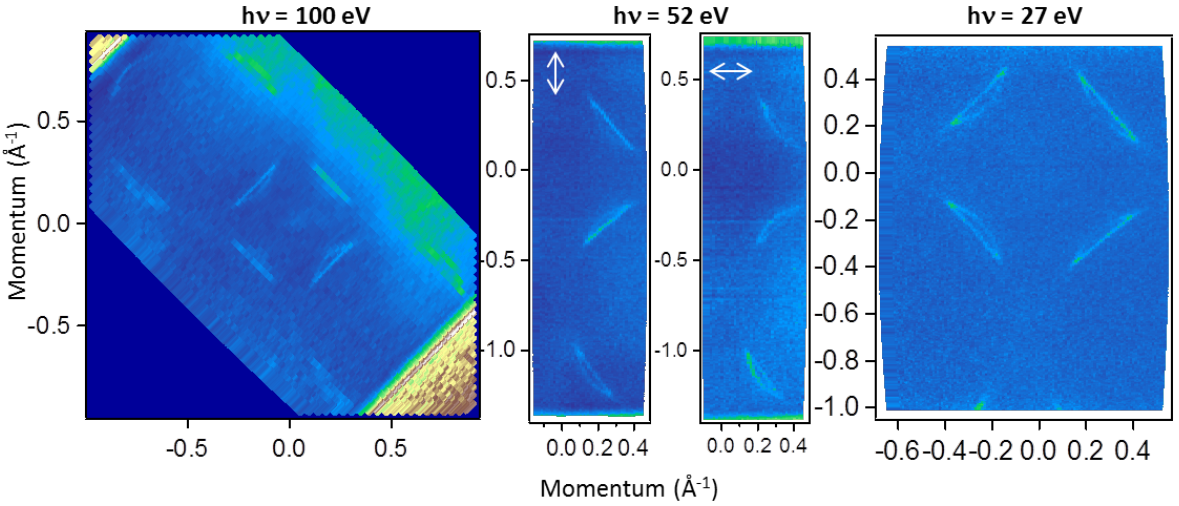

Supplementary Figure 6. Fermi surface maps of $EuMnBi_2$ for different excitation energies. White arrows indicate the direction of light polarization.

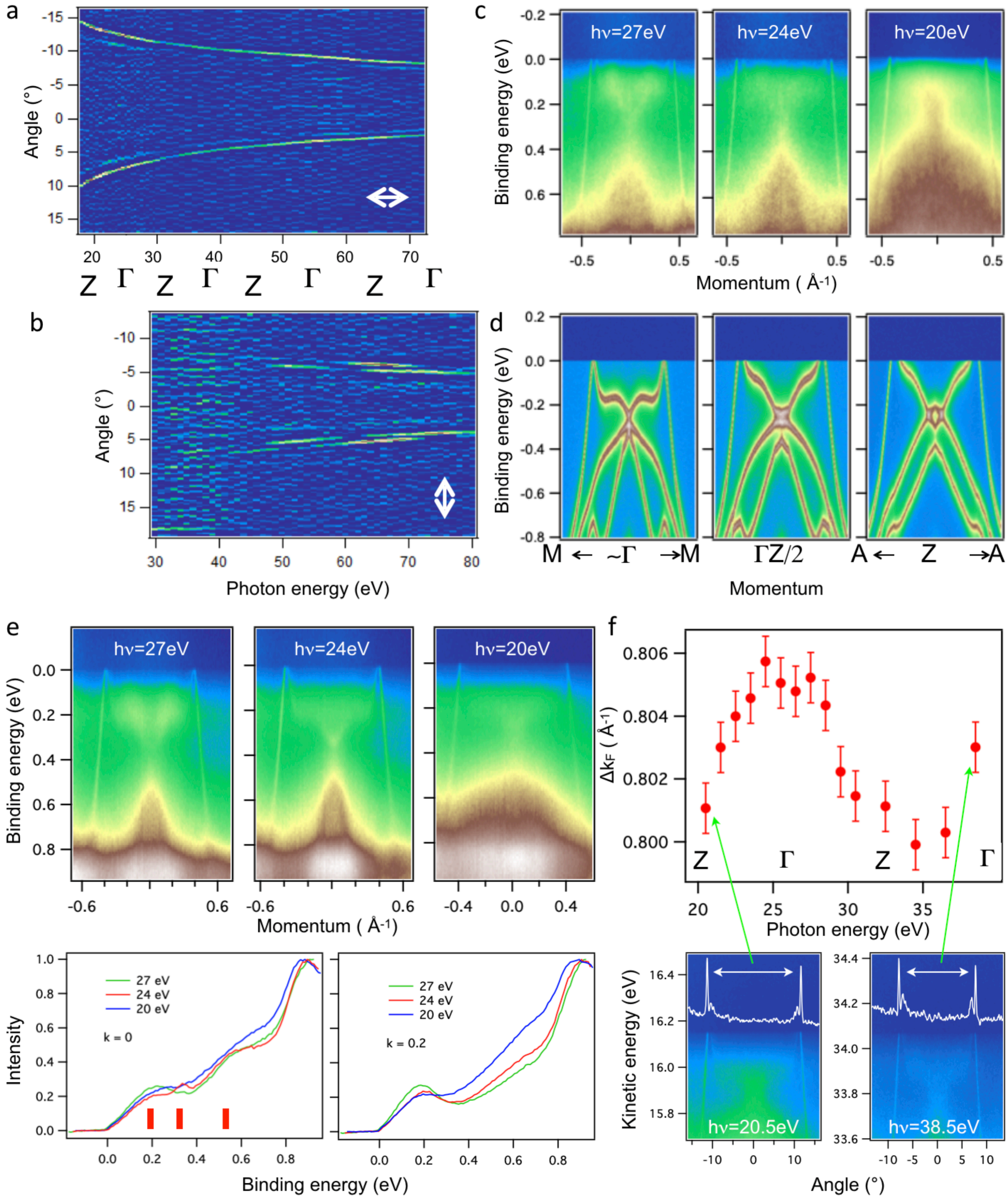

Supplementary Figure 7. $YbMnBi_2$: a) and b) second derivatives of the photon energy dependence of the integrated (40 meV) spectral weight close to the Fermi level for s- and p-experimental geometries along ΓM-direction. c) Examples of intensity maps taken at 27 (~Γ), 24 (~ΓZ/2) and 20 eV (~Z). d) Corresponding calculated bands. Fermi level is adjusted for better agreement with the experiment. The evolution of the 3D band with kz is seen. e) Similar to c) data recorded with better statistics from another sample together with the representative EDCs. Red bars show energy positions of the components. f) Variation of the distance between the steepest dispersions with photon energy. E_F-MDCs are shown together with two representative datasets as well.

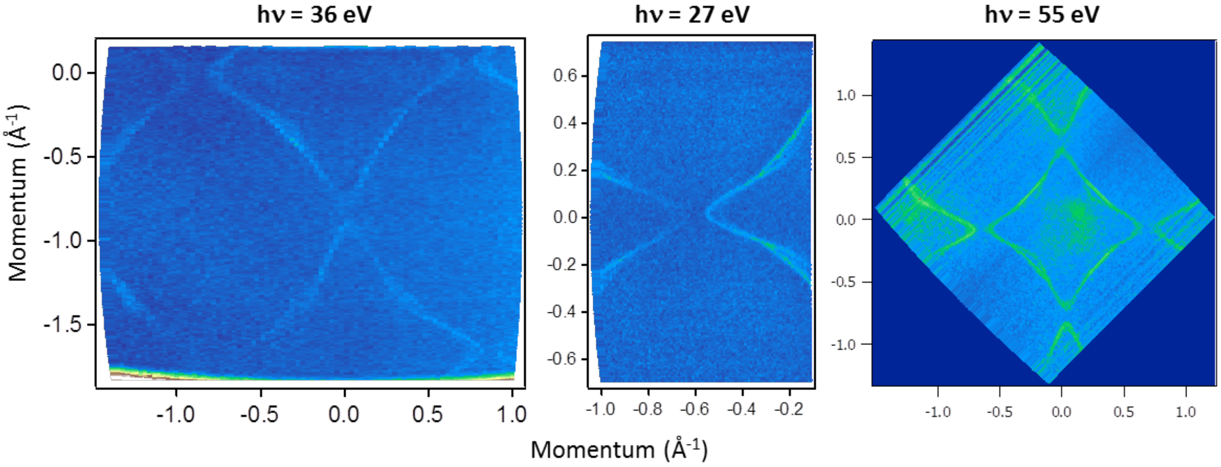

Supplementary Figure 8. Fermi surface maps of $YbMnBi_2$ for different excitation energies.

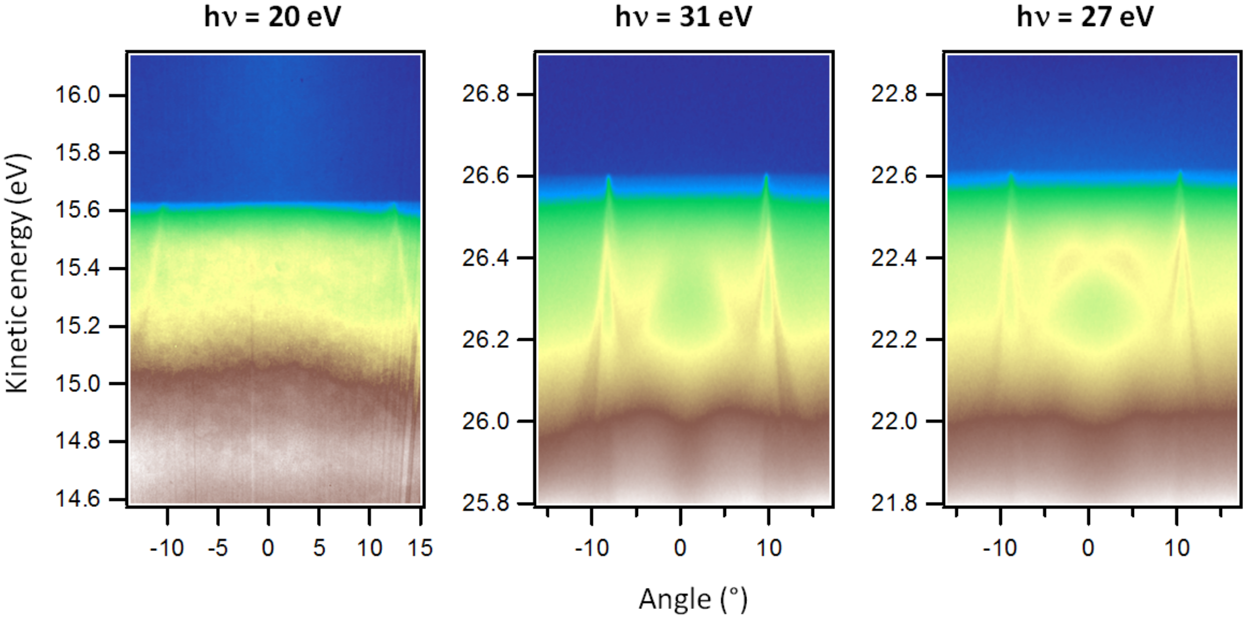

Supplementary Figure 9. $YbMnBi_2$: Momentum cuts through or close to the Weyl points at different photon energies.

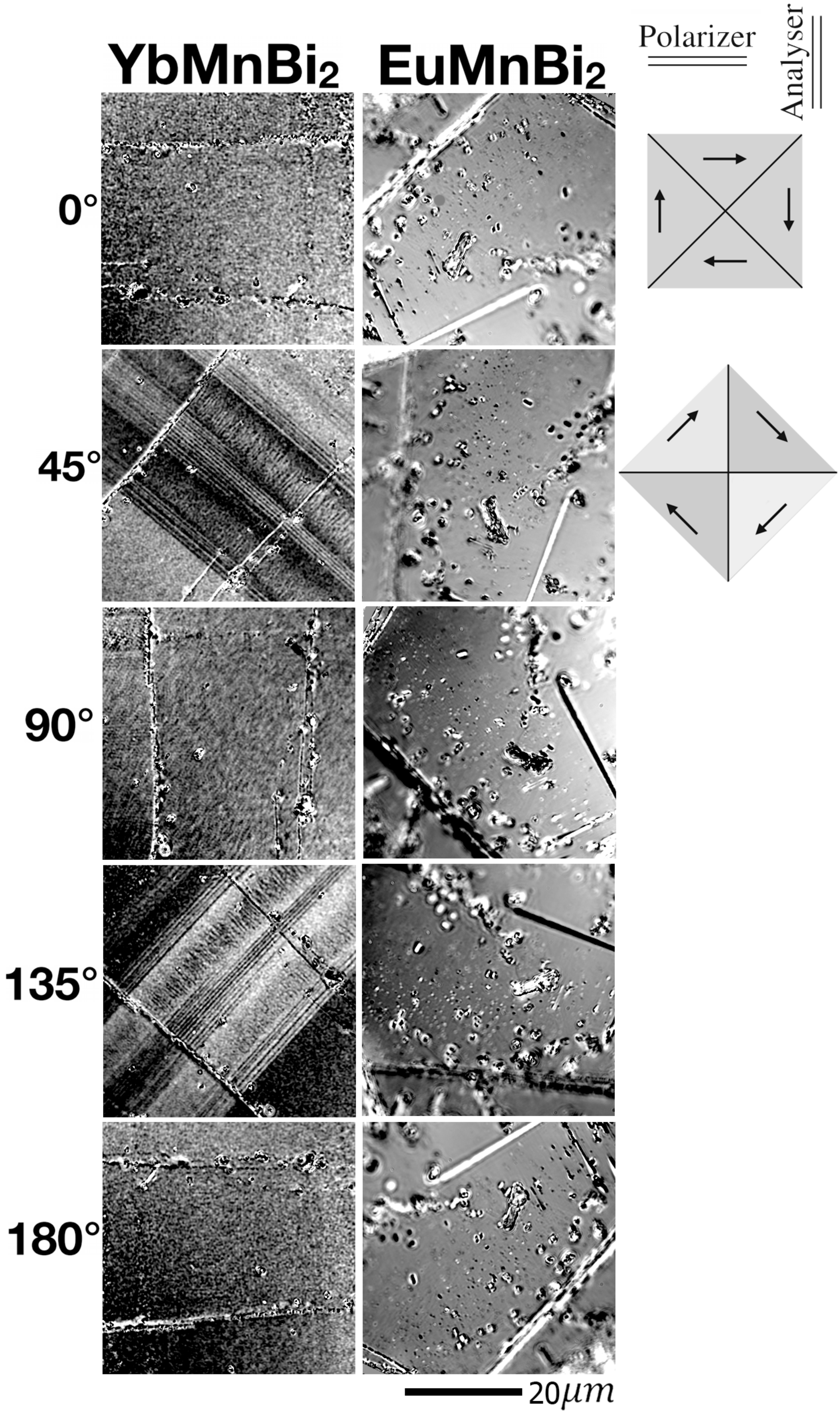

Supplementary Figure 10. Magneto-optical images of the $YbMnBi_2$ (left) and $EuMnBi_2$ (right) crystals, observed under conditions of the Voigt effect at perpendicular light incidence. A domain contrast can only be seen on the $YbMnBi_2$ material, proving the presence an absence of magnetic moment canting in $YbMnBi_2$ and $EuMnBi_2$, respectively (see text).

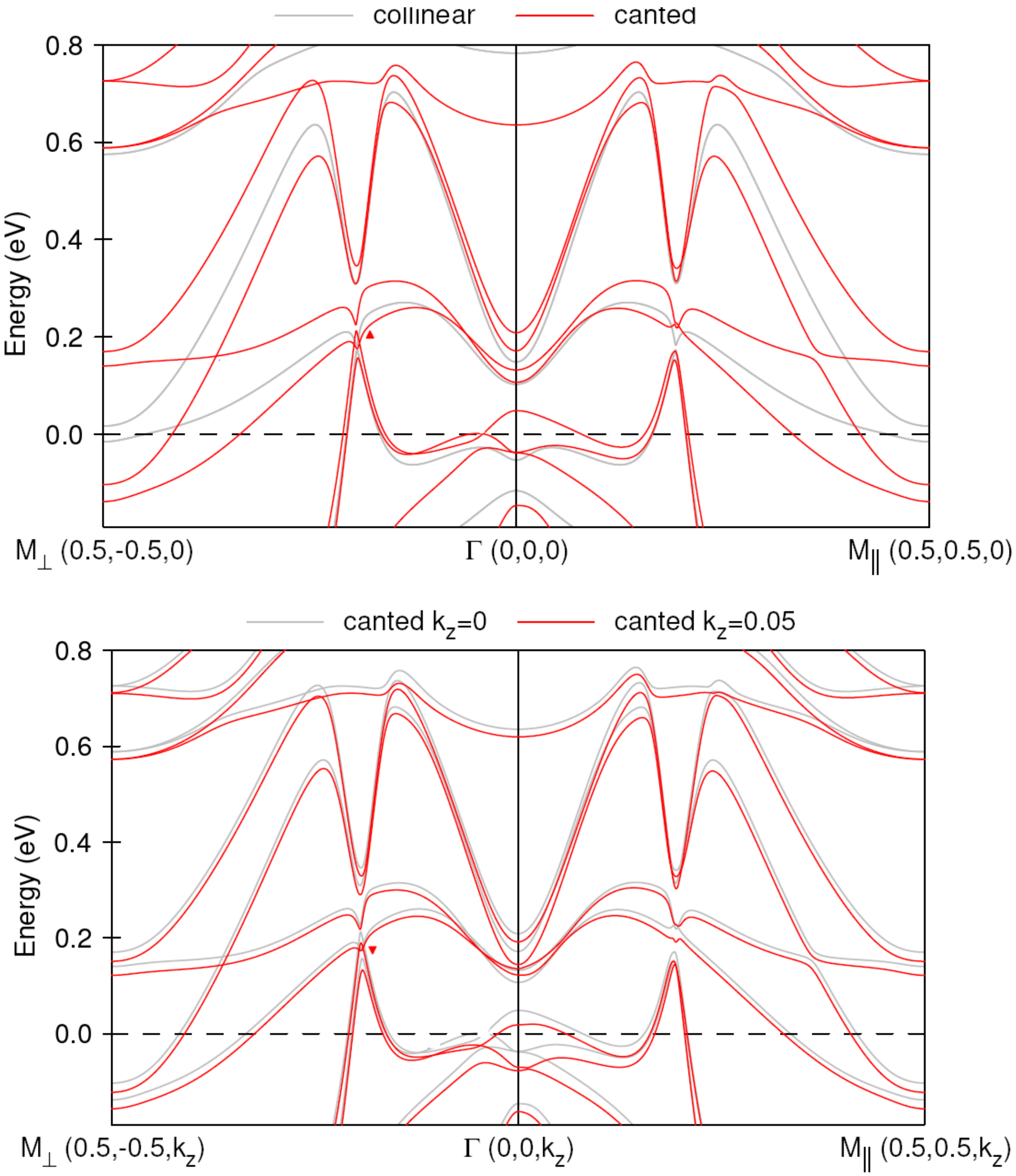

collinear
canted
0.8
0.6
0.4
0.2
0.0
Energy (eV)
M⊥ (0.5,-0.5,0)
Γ (0,0,0)
M|| (0.5,0.5,0)
canted kz=0
canted kz=0.05
0.8
0.6
0.4
0.2
0.0
Energy (eV)
M⊥ (0.5,-0.5,kz)
Γ (0,0,kz)
M|| (0.5,0.5,kz)

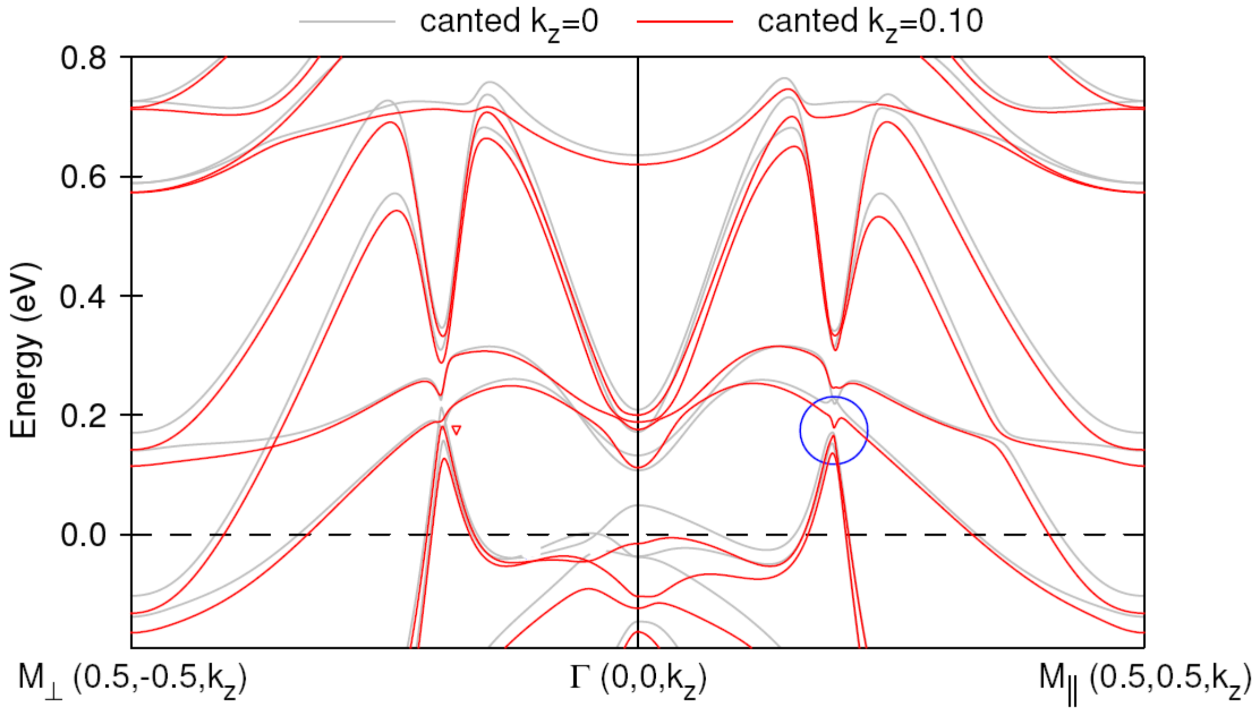
canted k_z=0
canted k_z=0.10
Energy (eV)
0.8
0.6
0.4
0.2
0.0
$M_\perp$ (0.5,-0.5,k_z)
Γ (0,0,k_z)
$M_\parallel$ (0.5,0.5,k_z)

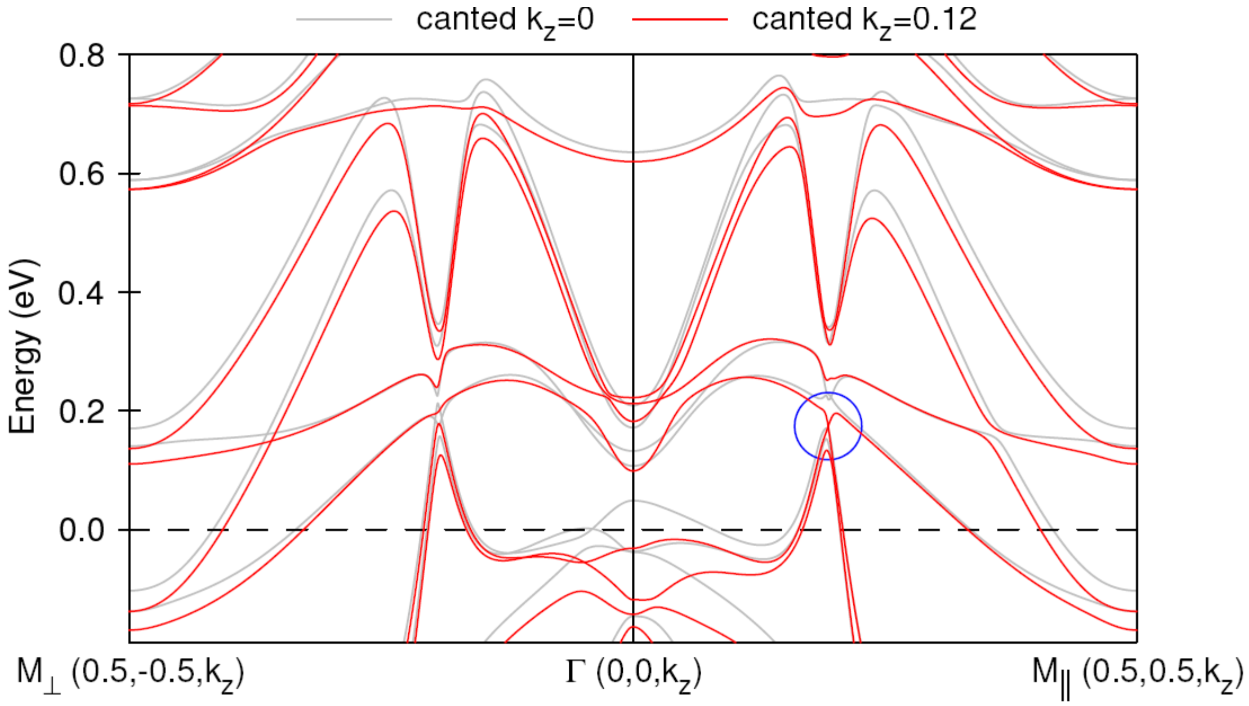
canted k_z=0
canted k_z=0.12
Energy (eV)
0.8
0.6
0.4
0.2
0.0
$M_\perp$ (0.5,-0.5,k_z)
Γ (0,0,k_z)
$M_\parallel$ (0.5,0.5,k_z)

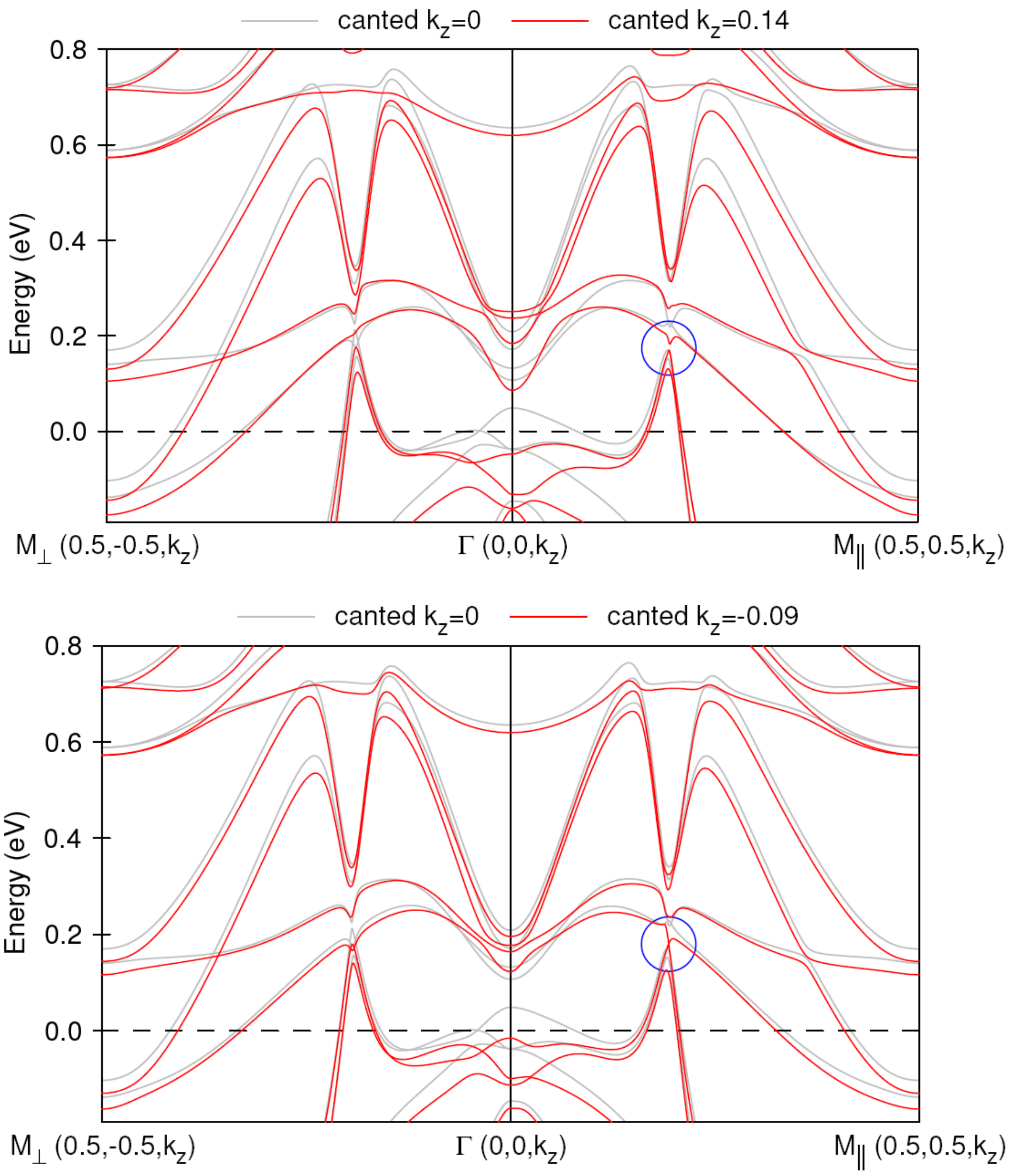

Supplementary Figure 11. Weyl crossings in high symmetry planes of $YbMnBi_2$. $\Gamma M_{\parallel}$ and $\Gamma M_{\perp}$ are directions along and perpendicular to the net magnetization respectively.

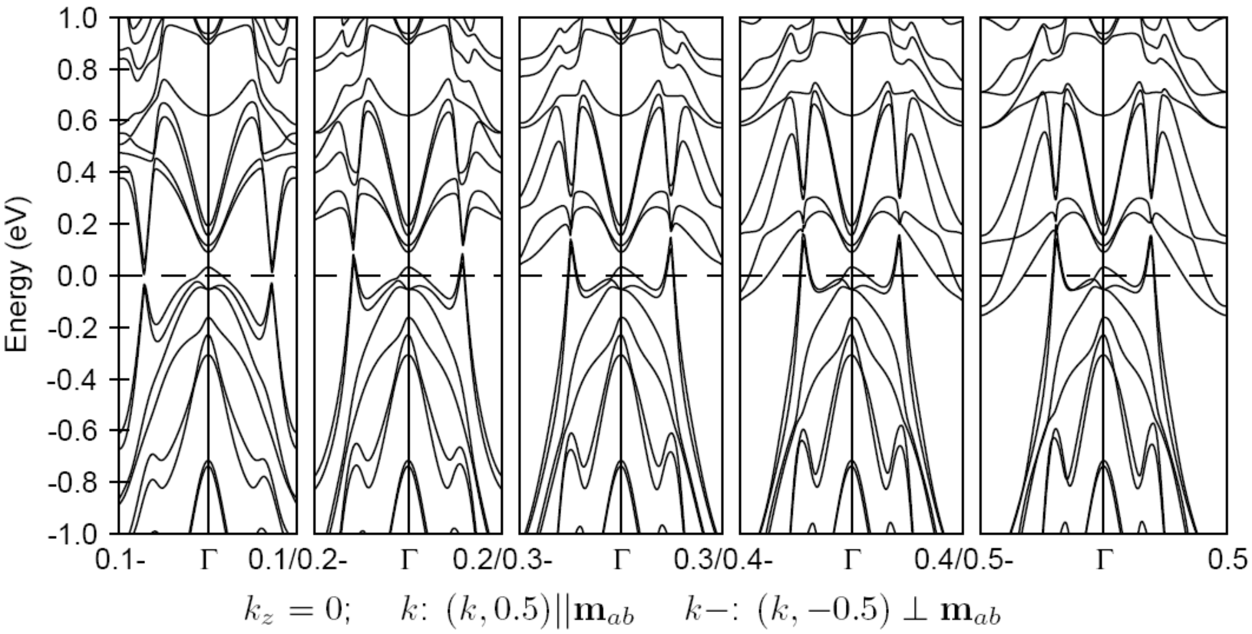

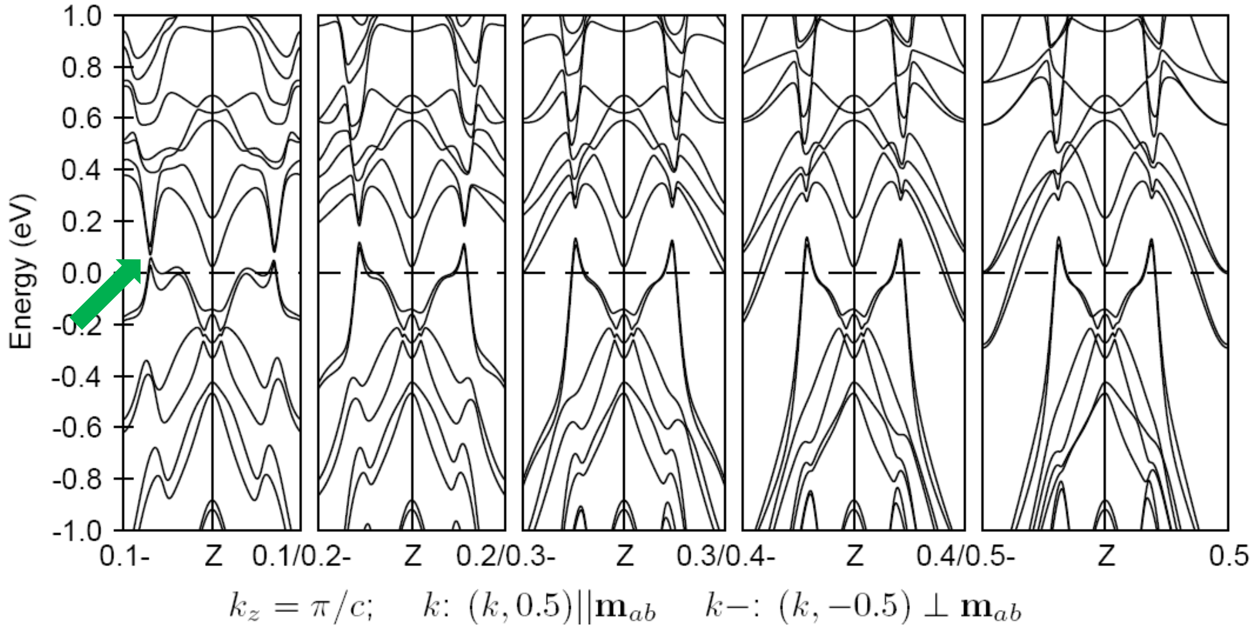

Supplementary Figure 12. $YbMnBi_2$: canting with $m_{ab}||(0.5, 0.5, 0)$. Green arrow shows the location where the gap is very small but finite (~8 meV).

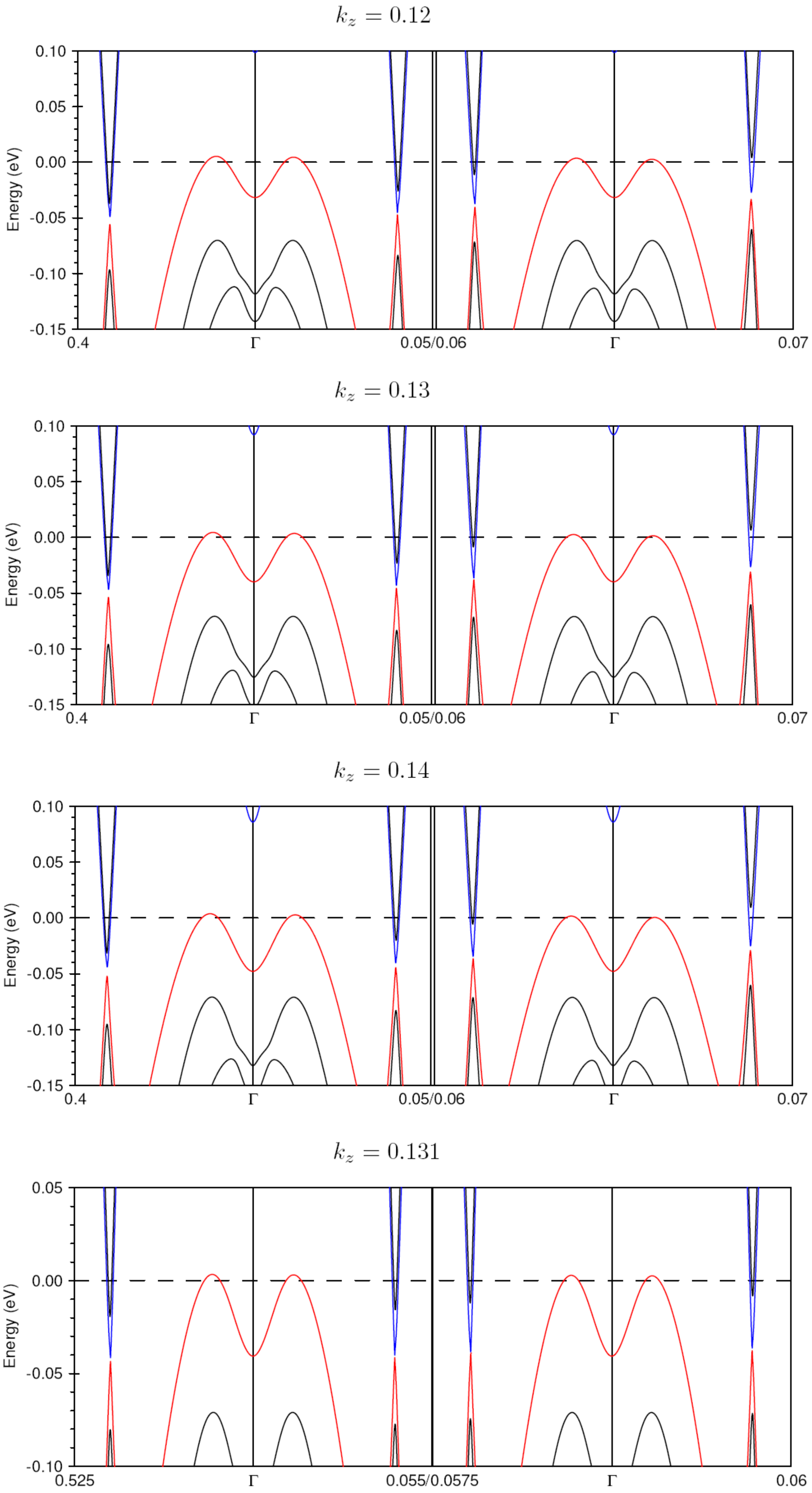

Supplementary Figure 13. Identification of the Weyl points in $YbMnBi_2$ corresponding to the experiment. Only the last panel shows the Weyl point itself when going from Γ to (0.0575, 0.5, 0.131). Other directions show very small gaps indicating a very anisotropic behavior close to the Weyl node.

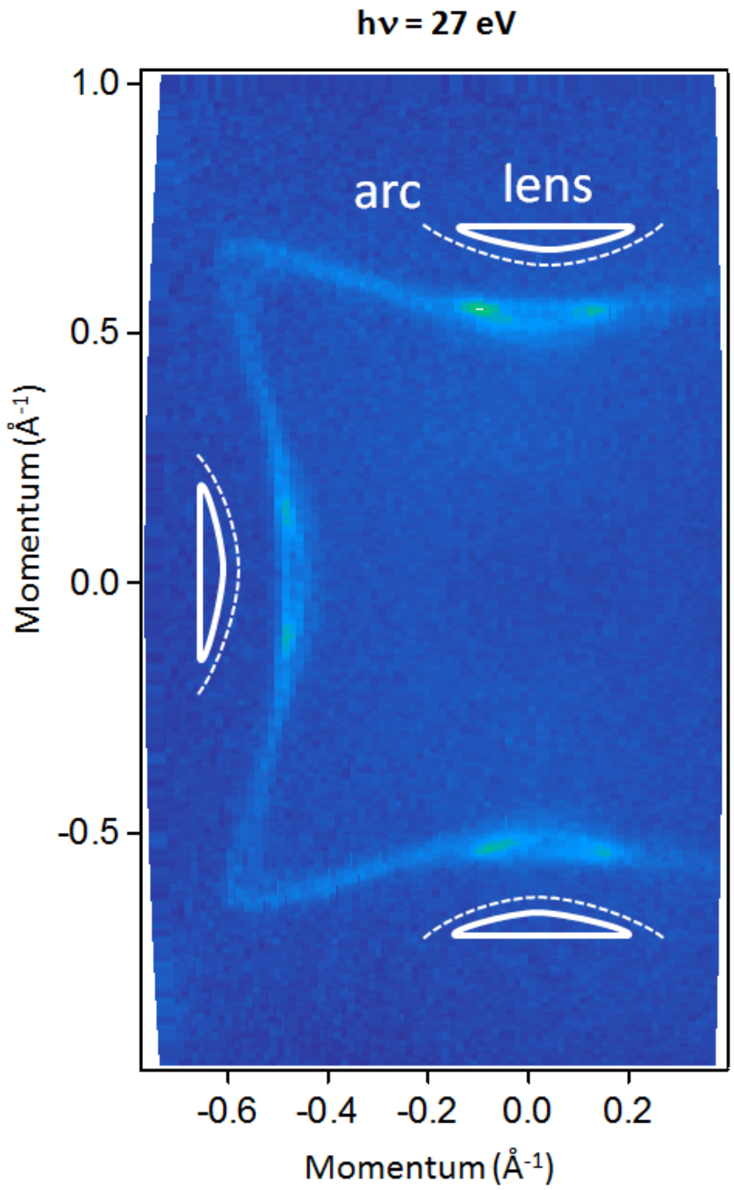

Supplementary Figure 14. Fermi surface map of $YbMnBi_2$ taken in particular geometry to distinguish the lenses from arcs. White lines are schematic guides to eye shifted from the experimental features. The sample has been oriented very precisely and the Fermi surface map has been recorded with the very small step.

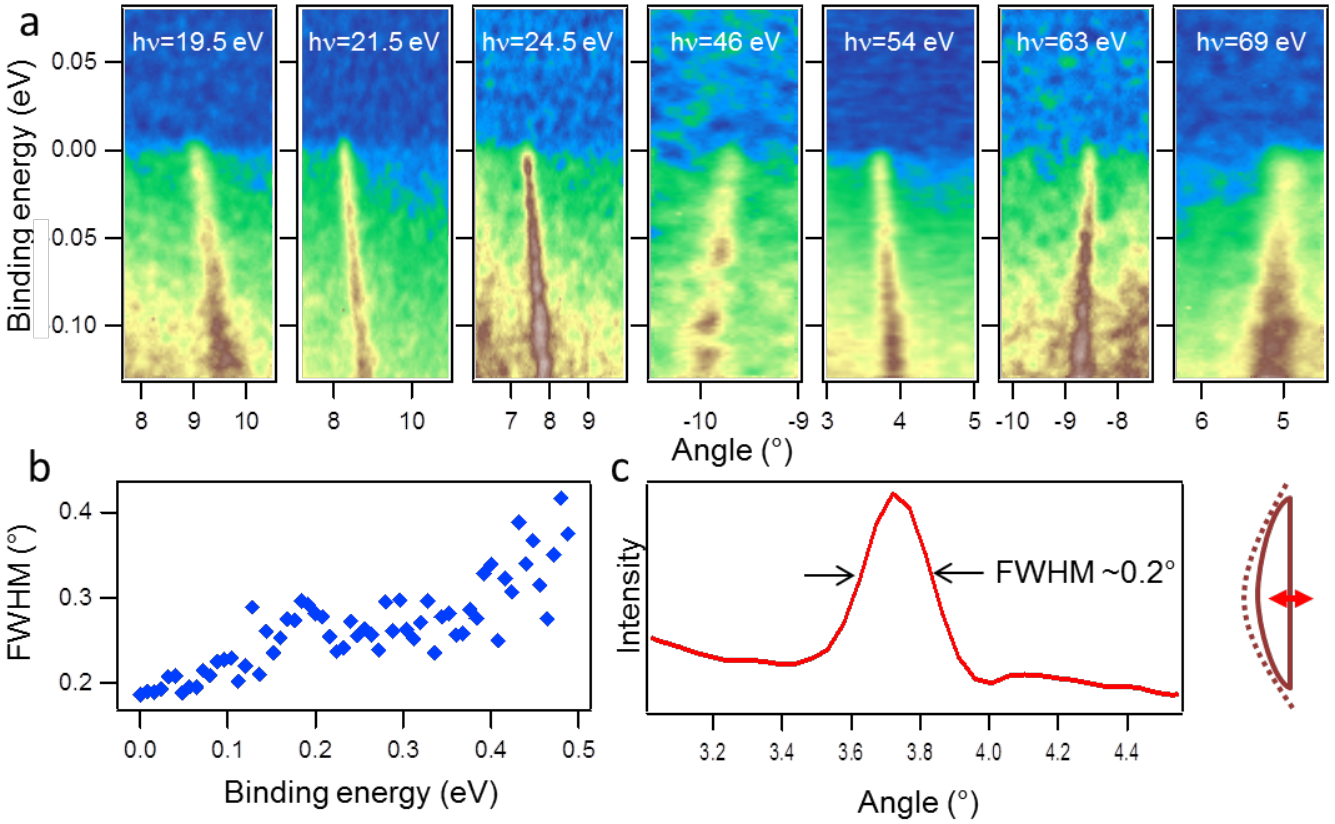

Supplementary Figure 15. $YbMnBi_2$: zoom in to the dispersion supporting the base of the lens as shown in the inset. a) photon energy dependence, b) typical FWHM of the k_F-MDC. c) typical k_F-MDC. There is always a single feature and its width in momentum is resolution limited corresponding to ~0.2°. Only at higher binding energies (~150 meV) the FWHM becomes larger, possibly indicating the presence of another feature.

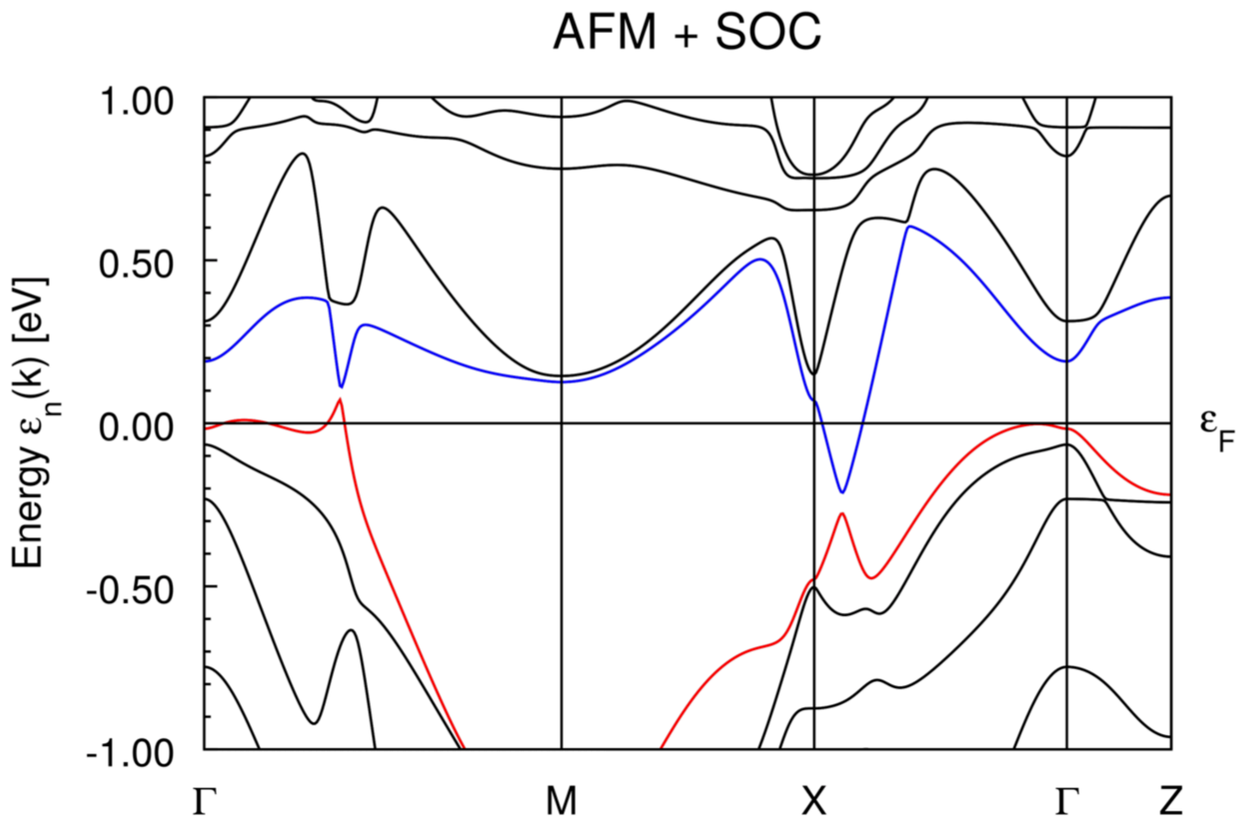

Supplementary Figure 16. FPLO band structure of $YbMnBi_2$: spin-orbit coupling and AFM of Mn atoms are included. No canting. Close agreement with LMTO is observed. The main difference is the position of the correlated Mn d-states (blue dispersion curve at M-point). The blue and red colored bands form the Weyl points after the introduction of canting.

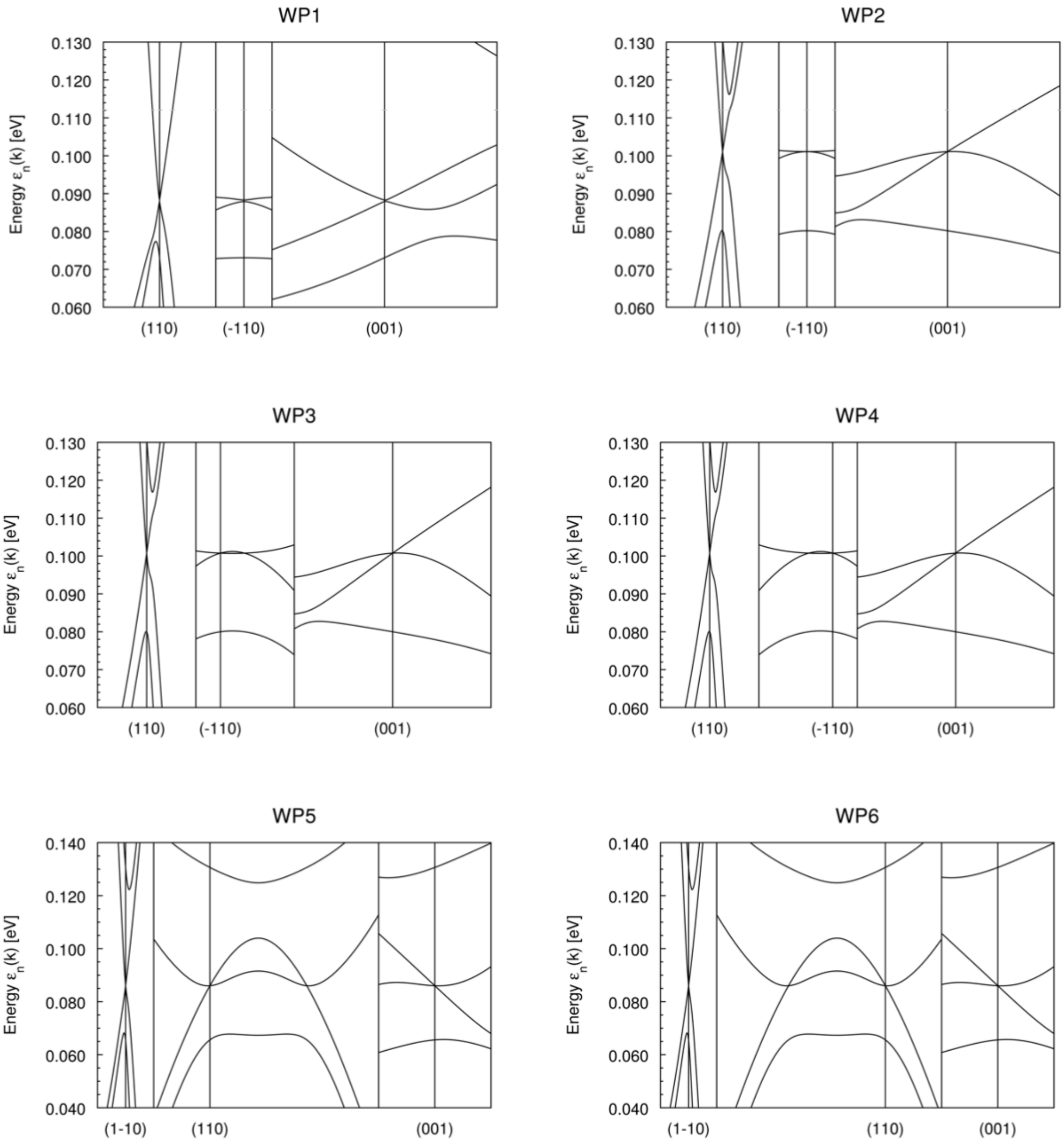

Supplementary Figure 17. FPLO band structure including canting. Dispersions along three perpendicular directions in the k-space are shown through all inequivalent Weyl points. The three directions are 1) perpendicular to the lens 2) along the lens and 3) along the z-direction. Note that the resolution of the WP position for WP1 is not perfect. Nevertheless, the Berry curvature proves that this is indeed a Weyl point. Type II Weyl character of the points #5 and #6 is clearly seen. Although it looks as if one of the bands forming WP5 and WP6 has a minimum at the WP it is not so. The minimum is slightly shifted off the exact WP position, thus establishing the Type II character.

Supplementary Tables

Supplementary Table 1. Photon energies at which high symmetry points are accessible considering that inner potential is 6.5 eV.

Symmetry point	Photon energy (eV)			
Γ	26	39	56	75
Z	20	32	47	65

Supplementary Table 2. Coordinates of the Weyl point as a function of canting angle.

Canting angle (°)	k_x (2π/a)	k_{z+} (2π/c)
10	0.194	0.120
5	0.193	0.070
2	0.193	0.040
1	0.193	0.027
0	0.193	0.015

Supplementary Table 3. Single crystal structure determination for $YbMnBi_2$, experimental data taken at 100 K.

Phase	$YbMnBi_2$
Symmetry	Tetragonal, *P4/nmm* (No. 129)
Cell Parameters (Å)	a = 4.478(1), c = 10.819(2)<br>α = β = ϒ = 90°
Wavelength (Å)	Mo Kα = 0.7107
V (Å^3)	216.94(3)
Z	1
Calculated Density (g cm^{-3})	4.94(1)
Formula Weight (g mol^{-1})	645.9
Absorption Coefficient (mm^{-1})	52.42
F_{000}	261.0
Independent/Observed Reflections	194/183
Data/Restraints/Parameters	194 / 0 / 23
Difference e- density (e/ Å^3)	+7.85 to -3.45
R_1 (all reflections)	0.044
R_1 Fo > 2σ(Fo))	0.0423
wR_2	0.108
R_{int}/R(σ)	0.0542/0.0304
GooF	1.34

Supplementary Notes

Supplementary Note 1.

In order to understand the complete 3D electronic structure of both materials we have recorded Fermi surface maps at different excitation photon energies. The results for $EuMnBi_2$ are shown in Fig. 6. Four lenses Fermi surface remains visible at different photon energies, which is not surprising since it originates from the 2D networks of Bi atoms. On the other hand, it can be due to the probing similar k_zs. As is seen from two middle panels, intensity distribution at the Fermi level only slightly changes as a function of light polarization. Intensity near the Γ-point does change and represents 3D band which also cross the Fermi level at particular k_z.

Since $EuMnBi_2$ did not show any evidence for the 3D Dirac or Weyl points in the following we deal with $YbMnBi_2$ only. In spite of very pronounced two-dimensionality of the features originated from the Bi-networks, we were able to detect clearly periodic patterns of intensity distribution along ΓM direction (Fig. 7 a,b,f) and determine photon energies which correspond to Γ and Z points in $YbMnBi_2$ (Table 1).

Intensity distributions at the energies corresponding to high-symmetry points are compared with calculations in panels c and d of Fig. 7. There is a qualitative agreement between two datasets confirming our assignment made in Table 1 and defined by the value of inner potential. Since the features near k=0 are blurred, we have recorded the similar data from another sample with better statistics (Fig. 7 e). While qualitatively the same due to finite kz-resolution of ARPES, the new dataset allows to demonstrate directly the kz-sensitivity of the features. The exemplary sets of EDCs clearly show the dispersion of the underlying components as well as different number of the features (e.g. marked by red bars).

The decisive evidence for the kz-dispersion comes from the dataset presented in Fig. 7 f. There we measured the k-separation of the steepest features at the Fermi level as the distance between the sharp peaks in the corresponding E_F-MDCs. This is illustrated with two extreme datasets taken using 20.5 and 38.5 eV photon energies. The distance obtained in this way from multiple datasets oscillates with photon energy between the values approximately corresponding to Γ and Z points.

We have recorded Fermi surface maps using different photon energies also for $YbMnBi_2$. The results are shown in Fig. 8. We emphasize here that the energies have been selected before the value of the inner potential has been found, i.e. before the assignment to G and Z points. The only criterion for the selection was the well-defined photoemission signal and sharpness of the features. At other photon energies we were not able to obtain a clear picture of the Fermi surface. Now it can be explained in terms of two regions of kz identified above: at those kz which correspond to either minimal gaps or Weyl crossings the kz dispersion is naturally weaker.

We have also checked that the lifting of the degeneracy is observed at different photon energies to exclude its artificial origin. Corresponding data sets are shown in Fig. 9. It is seen that the linear features dispersing from higher binding energies split and only one pair reaches the Fermi level for both, positive and negative angles.

Supplementary Note 2.

As discussed in the main text, magnetic contrast can only be seen on the surface of the $YbMnBi_2$ sample by employing the Voigt effect at perpendicular light incidence in an optical polarization microscope, while no Voigt domain contrast shows up on the $EuMnBi_2$ crystal.

Typical for the Voigt effect is its 90° symmetry on rotating the sample: the domains show up with maximum contrast if their axes of magnetization are at 90° relative to each other and if they are aligned at 45° to polarization axis of the illuminating light (see sketches in Fig. 10). The contrast disappears if the sample is rotated by 45° and it shows up

again with maximum, but now inverted contrast after a 90°rotation. For our $YbMnBi_2$ sample this typical contrast symmetry is revealed as shown in the left column of Fig. 10.

For our $EuMnBi_2$ crystal, no Voigt contrast is observed at perpendicular incidence. This indicates an antiferromagnetic alignment of magnetic moments with no in-plane components of the occupied crystallographic axis. Also a canting of moments along a single axis can be excluded, as in this case the brightness of the images should at least reveal the 90° symmetry of the Voigt effect. This is not the case by comparing the images in the right column of Fig. 10.

Note that the external magnetic fields, available in our microscope setup, are too small to reorient the magnetization of the $YbMnBi_2$ crystal. Therefore the conventional difference image technique [1] with reference image in saturated state could not be applied and all shown images are difference images with the analyser been opened (in respect to polarizer) in opposite direction for reference image, what inverts the domain contrast. However, this technique provides less contrast to be reasonably seen with microscope, configured for longitudinal Kerr effect (not shown), and, unfortunately, the contrast enhancement upon analyser rotation cannot be employed together with selective-sensitivity technique [2], what limits the domain observation in our samples to only Voigt effect.

Supplementary Note 3.

In order to explain the splitting of the bulk bands in $YbMnBi_2$ seen experimentally we have carried out the calculations with canting. Canting angle is 10°. It produces net FM magnetization along (1, 1, 0). The results are shown in Fig. 11.

As is seen from the calculations, there are two closely separated crossings of the singly degenerate bands on the $\Gamma M_{\perp}$ direction, but these are not 3D Weyl points, as evolution with kz shows. These crossings make a loop in the vertical plane. These two symmetry related loops are shown in Fig. 2c of the main text. On the $\Gamma M_{\parallel}$ direction, in contrast, a pair of true 3D Weyl points is observed at kz=0.12 and kz=-0.09. Away from vertical ZΓM planes all crossings become avoided. The locations of 3D Weyl points are symmetrical with respect to earlier detected 3D Dirac point (kz=0.015) for collinear configuration of spins, implying that this pair of Weyl points is created by the time-reversal symmetry breaking (canting). In order to test this, we have carried out the calculations for different values of canting angle. Indeed, the distance between the Weyl point and initial 3D Dirac point decreased with decreasing the canting angle (Table 2).

We have also scanned other portions of BZ and found another set of Weyl points, lying away from high-symmetry planes and directions. First, in Fig. 12 we show the radial cuts from Γ-point towards the Fermi surface contour for kz=0 and kz=π/c.

As is seen, the very small gap is detected in the ZAR-plane signaling the proximity to a Weyl point. Further scanning of momentum space resulted in identification of Weyl points with very anisotropic dispersions at kz=0.131 (see Fig. 13). The Weyl point is found at (0.394,0.045, 0.131). The kx and ky of this position corresponds to the point where the hole-like lens is connected to electron-like pocket.

Other symmetry related Weyl points are at (0.394,-0.045,0.131), (0.045, 0.394,0.131) and (-0.045, -0.394,0.131). As follows from the presented data, there are two k_z values which define the k_x-k_y planes where Fermi surface may look continuous in the experiment: $k_z=\pi/c$ where the gaps are very small and k_z~0.1 where the true 3D Weyl points are observed.

Supplementary References

[1] F. Schmidt, W. Rave, and A. Hubert, IEEE Trans. Magn. 21, 1596 (1985)

[2] I. V. Soldatov and R. Schäfer, Rev. Sci. Instrum. **88**, 073701 (2017).
